# 3D Spheroid Cultures of Stem Cells and Exosome Applications for Cartilage Repair

**DOI:** 10.3390/life12070939

**Published:** 2022-06-22

**Authors:** Seung Yeon Lee, Jin Woo Lee

**Affiliations:** 1 Department of Molecular Medicine, College of Medicine, Gachon University, 155, Gaetbeol-ro, Yeonsu-ku, Incheon 21999, Korea; totoro218@hanmail.net; 2 Department of Health Sciences and Technology, GAIHST, Gachon University, 155, Gaetbeol-ro, Yeonsu-ku, Incheon 21999, Korea

**Keywords:** spheroid, 3D culture, exosome, mesenchymal stem cells, cartilage

## Abstract

Cartilage is a connective tissue that constitutes the structure of the body and consists of chondrocytes that produce considerable collagenous extracellular matrix and plentiful ground substances, such as proteoglycan and elastin fibers. Self-repair is difficult when the cartilage is damaged because of insufficient blood supply, low cellularity, and limited progenitor cell numbers. Therefore, three-dimensional (3D) culture systems, including pellet culture, hanging droplets, liquid overlays, self-injury, and spinner culture, have attracted attention. In particular, 3D spheroid culture strategies can enhance the yield of exosome production of mesenchymal stem cells (MSCs) when compared to two-dimensional culture, and can improve cellular restorative function by enhancing the paracrine effects of MSCs. Exosomes are membrane-bound extracellular vesicles, which are intercellular communication systems that carry RNAs and proteins. Information transfer affects the phenotype of recipient cells. MSC-derived exosomes can facilitate cartilage repair by promoting chondrogenic differentiation and proliferation. In this article, we reviewed recent major advances in the application of 3D culture techniques, cartilage regeneration with stem cells using 3D spheroid culture system, the effect of exosomes on chondrogenic differentiation, and chondrogenic-specific markers related to stem cell derived exosomes. Furthermore, the utilization of MSC-derived exosomes to enhance chondrogenic differentiation for osteoarthritis is discussed. If more mechanistic studies at the molecular level are conducted, MSC-spheroid-derived exosomes will supply a better therapeutic option to improve osteoarthritis.

## 1. Introduction

All cells in the body exist in a three-dimensional (3D) environment, and their phenotypes and functions are greatly dependent on elaborate interactions with neighboring cells, proteins, and extracellular matrix (ECM) [1]. In two-dimensional (2D) cell cultures, however, cell-to-cell and cell-to-ECM interactions are reduced, and cell responsiveness is limited. Thus, toxicity tests of materials and substances on 2D cell cultures is not fully predictive of what may be assumed in vivo [2,3]. Therefore, 3D cell cultures are widely used in the study of cancer cells, cell differentiation, intracellular interactions, material toxicity, and efficacy of potential drugs [3].

Cell biology-based 3D culture systems are classified as organoids produced by differentiating stem cells in vitro into multicellular tissues and spheroids formed by aggregating single cells derived from human organs [4]. In particular, intercellular adhesion and differentiation for forming spheroids are divided into three stages: (1) incomplete cell aggregation by the binding of integrin and ECM, (2) the expression and accumulation of cadherin, and (3) hemophilic interaction of epithelial cadherin through cadherin–cadherin binding [5]. A simple and reproducible technique for generating spheroids is essential for research.

Scaffold-free and scaffold-based culture systems made of natural or synthetic materials are the two broad categories of 3D culture approaches. There are several scaffold-based 3D culture methods, which can be roughly divided into two approaches: hydrogels and solid scaffolds [6]. Encapsulating cells in a hydrogel with a loose scaffold frame of a crosslinked natural base material, such as collagen, fibrin, hyaluronic acid, or agarose, with a high water content, is a popular option for 3D culture [7]. Hydrogels can be designed to support specific types of cell growth and function by trapping cells in an artificial ECM protein environment or allowing cells to migrate from the gel’s surface into its interior [8,9,10]. Seeding cells into a solid scaffold, on the other hand, provides 3D space to support the cells, allowing them to form natural 3D tissue-like structures. The ability of solid scaffold-based technologies to support 3D cultures and produce organized arrangements of cells in vitro in a controllable and reproducible manner, using methods appropriate for routine use, is an advantage [6].

**Figure 1:**
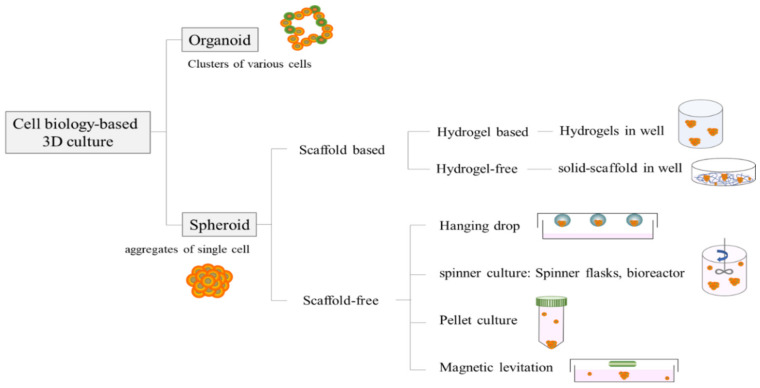
3D culture techniques used in publications to study stem cell differentiation.

Over the past decade, many scaffold-free-based spherical manufacturing methods have been reported. The hanging drop technique was used to create a 3D microenvironment niche for tissue development; it is a method in which cells gather at the drop’s tip and spontaneously aggregate to form spheroids [11]. Continuous agitation is used in spinner culture by gently agitating the cell suspension with an optimal cell density, rotating the chamber, or perfusing the culture medium, through the scaffold with a pump system. Therefore, this method is suitable for intensive cell expansion and the large-scale production of biomolecules, such as growth factors and antibodies [12]. In pellet cultures, cells are collected using gravity or centrifugal force. This method has the disadvantage of causing hypoxia in the core as it creates large spheroids with a diameter of more than 500 µm [13]. However, in general, a hypoxic environment is suitable for bone regeneration studies because it stimulates chondrocyte differentiation and mesenchymal stem cell cartilage formation [14]. Magnetic cell levitation is a new method for spheroid generation that has recently been developed. Magnetic levitation involves preloading cells with magnetic nanoparticles and encouraging them to form multicellular spheroids within hours using an external magnetic field. Because of their magnetic properties, the resulting spheroids can be easily manipulated and confer benefits such as cell tracking and imaging [15]. The general 3D culture techniques used to study stem cell differentiation are summarized in Figure 1.

## 2. Chondrogenic Differentiation of Stem Cells

**Figure 2:**
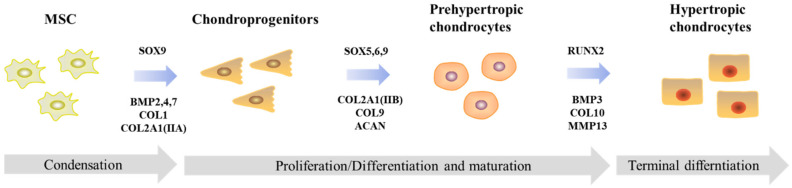
Schematic representation of chondrogenic differentiation of mesenchymal stem cells. MSC, mesenchymal stem cell; SOX5,6,9, SRY-box transcription factor 5/6/9; BMP2,4,7, bone morphogenetic protein 2,4,7; COL1, collagen type I alpha 1; COL2A1, collagen type II; COL9, collagen type IX alpha 1; ACAN, aggrecan; RUNX2, RUNX family transcription factor 2; BMP3, bone morphogenetic protein 3; COL10, collagen type X alpha 1; MMP13, matrix metallopeptidase 13.

Cartilage is a connective tissue that acts as a pressure buffer and is necessary for joint movement protection [16]. Chondrocytes are the cartilage’s single cell type and demonstrate distinctive features, such as being metabolically active to maintain ECM turnover by synthesizing glycoproteins, collagens, proteoglycans, and hyaluronan [17]. Chondrocytes differentiate into proliferating, pre-hypertrophic, and hypertrophic chondrocytes during chondrogenesis. Finally, the hypertrophic chondrocytes are subjected to apoptosis and are replaced by bone tissue. Chondrogenic differentiation of MSCs is a complex developmental process governed by several factors such as growth factors, signal pathways, and transcription factors, all of which must be activated in a specific spatiotemporal sequence for proper chondrocyte formation (Figure 2) [18,19]. SRY-box transcription factor (SOX)9 promotes mesenchymal cell differentiation into chondrocytes by upregulating early chondrogenic genes, including type II collagen [Col2a1(IIa)], type IX collagen (Col 9a), type XI collagen (Col11a2), and aggrecan [20]. The SOX transcription factor family (SOX5, SOX6, and SOX9) plays various roles during chondrogenic differentiation, with SOX9 being the primary determinant during the early stages of chondrogenesis [21]. Runt-related transcription factor 2 (RUNX2) is crucial for mediating chondrocyte maturation and regulating the expression of type X collagen (Col10a1) in hypertrophic chondrocytes, therefore enhancing endochondral ossification [16]. Several bone morphogenetic proteins (BMPs) play important roles in cartilage homeostasis. BMP2, BMP4, and BMP7 can induce chondrogenic differentiation by regulating SOX9 expression and can stimulate endochondral ossification by regulating RUNX2 transcription [16]. Moreover, BMP3 promotes the maturation of terminal hypertrophic chondrocytes [22]. Matrix metallopeptidase 13 (MMP13) is an enzyme that regulates cartilage matrix degradation, which occurs prior to mineralization by osteoblasts, is required for the formation of the bone marrow space, and promotes vascular invasion. This produces the cells that make up bone marrow. MMP13, a matrix-degrading enzyme, is expressed only by the most terminally differentiated hypertrophic chondrocytes [23]. However, chondrocytes cultured in vitro have a highly susceptible growth and differentiation control mechanism, which can easily lead to cell differentiation and aging [16]. 3D culture can be an effective method for regulating the mechanisms of chondrogenic differentiation and cartilage-related therapeutic strategies. Table 1 summarizes the 3D culture studies related to fostering chondrogenesis using each 3D culture method.

## 3. Application of 3D Spheroid Culture System to Chondrogenic Differentiation of Stem Cells

### 3.1. Cartilage Regeneration with Stem Cells Using 3D Spheroid Culture System

When articular cartilage is injured, it has a low regenerative capacity due to a lack of blood supply, a limited number of progenitor cells, and low cellularity [37]. Cartilage repair is also related to OA, which requires hip replacement surgery with a total hip implant [38]. Despite the fact that controlling cell shape and positioning is a promising approach for cartilage repair, functional cell types and biomaterials are lacking [39]. Several studies to date have demonstrated that stem cells or progenitor cells derived from human fetal tissue are a promising cell source for cell therapy and tissue engineering [40]. Because of its high potential for biocompatibility compared to single cells, 3D culture technology has been steadily researched since the 1990s and has recently evolved into 3D spheroids. Spheroids are thought to better reflect cell organization than 2D cell cultures, and stem cell spheroids have been extensively studied in therapeutic transplantation.

Unlike a conventional single cell culture, a 3D cell culture creates an environment similar to a living body in vitro. As a result, all cells form a cell culture model grow well and allow for interactions with the created environment [41]. Yoon et al. [24] demonstrated that spheroid culture can be used to achieve large-scale in vitro chondrogenic differentiation of human adipose-derived mesenchymal stem cells (hADSCs) and subsequent in vivo cartilage formation. Due to the lack of vasculature, articular cartilage function is specifically maintained in a low-oxygen environment throughout its life. The oxygenation gradients within this avascular tissue were estimated to drop from 6–10% at the surface to below 1% in the deepest layers, indicating that a physiologically hypoxic microenvironment is required for the maintenance of articular cartilage homeostasis [42]. Because of oxygen diffusion limitations, the cells beneath the surface of spheroids are generally subjected to mild hypoxia. This environment significantly increases the expression of hypoxia-inducible factor-1 alpha (HIF-1) in spheroid cells. HIF-1 is a component of cells’ inherent ability to respond to low-oxygen environments by stimulating the production of SOX-9, a chondrogenic transcription factor [43]. Hypoxic conditions appear to provide a more favorable microenvironment for hADSC chondrogenesis. The authors discovered that a hypoxic environment in spheroids increased HIF-1 expression by activating p38 and AKT, resulting in enhanced chondrogenesis in hADSCs cultured in spheroids.

Pellet culture is the most popular 3D culture model [44]. In a study by Tsvetkova et al. [30], spheroids derived from adipose tissue MSCs contained the most ECM and a high concentration of glycosaminoglycans. Chondrocytes produce a matrix rich in glycosaminoglycans. Type II collagen is primarily produced by chondrocytes and adipose tissue MSCs (to a lesser extent). In addition, the authors [32] showed that functional matrix accumulation, but not chondrogenic gene expression, correlated highly with aggregate morphology as early as day seven, highlighting a discrepancy between chondrogenic phenotype and gene expression. MSC aggregate size was much earlier correlated with chondrogenic synthetic activity than the shape of MSC that had aggregated, which became significantly correlated by day 14. There was no overlap with markers for hypertrophic chondrocytes such as alkaline phosphatase (ALP) and collagen type X (COLX), and during embryonic development, ITM2A expression preceded ALP expression [45]. This finding suggests that ITM2A is involved in early chondrogenesis. The authors discovered that ITM2A expression profiles differ in human primary mesenchymal stem cells derived from bone marrow and adipose tissue, and that its regulation during in vitro chondrogenesis indicates that this gene may be involved in the blocked of chondrogenesis initiation [33]. SOX proteins are required for chondrogenesis, according to several lines of evidence, both in vitro and in vivo. Except for hypertrophic chondrocytes, SOX9 is expressed in all chondroprogenitors and chondrocytes [46]. In vitro, SOX9 binds to and activates chondrocyte-specific increaser elements in COL2A1, COL9A1, COL11A2, and aggrecan (ACAN) [47,48]. Ikeda et al. [35] were the first to show that SOX9 promotes the expression of SOX5 and SOX6. Furthermore, the authors discovered that the SOX trio induced cartilage-specific genes, Chondromodulin 1 and matrilin 3, that did not belong to collagens or proteoglycans. Chondromodulin 1 and matrilin 3 expression are known to be cartilage-specific [49].

The traditional hanging drop method of forming 3D cell spheroids has been suggested to improve therapeutic potential and has been shown to elicit an anti-inflammatory effect in MSCs [50]. The pellet size was optimized by Jisheng Ran et al. [26]. Pellets containing 40,000 cartilage stem/progenitor cells (CSPCs) were found to have the most abundant cartilage matrix deposition and the highest mRNA expression levels of SOX9, ACAN, and COL2A1 during chondrogenic induction when compared to pellets containing 10,000, 100,000, or 200,000 CSPCs. According to Sridharan et al. [27], the most important parameter during spheroid formation is medium composition. Although several groups have investigated insulin-like growth factor for chondrogenesis, this was the first study to demonstrate that insulin-like growth factor can be used as a key formulation in stem cell spheroids.

Iron oxide magnetic nanoparticles have been investigated as agents for cell magnetic separation, magnetic resonance imaging, drug delivery, tracking, and cellular therapy targeting [25]. The authors demonstrated that a levitated culture improved spheroid formation and cell mobility, as well as preserved or enhanced the “stemness” of adipose-derived mesenchymal stem cell-magnetic nanoparticles (ADSC-MNP), increasing proliferation, adipogenesis, chondrogenesis, and osteogenesis.

Scaffold-based models better mimic cell-to-ECM interactions, whereas spheres of a certain size are more susceptible to cellular and physiological gradients [51]. Multicellular MSC spheroids have an increased chondrogenic ability and low fibrosis, making them ideal for hyaline-like cartilage regeneration. Nevertheless, due to the blocked cell surfaces in spheroids exposed to DNA/vectors, efficient gene transfection is difficult to achieve, as most rely heavily on cell–substrate interactions. As a result, the authors described that their poly (l-glutamic acid)-based porous scaffold possessing the tunable inner surfaces enable subsequent in situ spheroid formation as well as the sequential cell–scaffold attachment and detachment [32].

Hydrogels using micro-mold technology have recently emerged as spheroid culture alternatives due to their automation capacity and long-term culture for differentiation assays [52]. The micro-molded non-adhesive hydrogel allowed seed cell suspension with single pi-petting, significantly reducing technical errors reflected in the spheroid size and shape homogeneity [53]. Hydrogels are networks formed by intermolecular or interfibrillar crosslinks from dilute polymer chains with a specific structure and properties. Natural hydrogels, such as fibrin, collagen, or Matrigel, have superior biocompatibility, natural adhesive properties, and many physiological properties for cell functions, resulting in controlled proliferation or differentiation, high cell viability, and frequently a cell phenotype seen in vivo [7]. Alginate, which is isolated from the cell wall of brown algae, is another natural hydrogel. The mechanical properties and the rapid degradation of alginate hydrogel limit its use in 3D culture process, but it has been used in 3D-printed scaffolds for the regeneration of specialized tissues such as vascular tissue, cartilage, and bone [54,55]. Nemeth et al. [28] first reported that PEG–GelMA–HA nano-patterned hydrogels stimulate chondrogenic differentiation of dental pulp stem cells (DPSCs). Their scaffolds created a nano-topographically defined 3D environment with 500 nm grooves. This 3D nano-topography environment enhanced a formation of the sphere, mimicking a natural roundness of the chondrocytes on the unattached environment. These authors discovered that differentiated DPSCs, those that were cultured on PEG–GelMA–HA nano-patterned scaffolds, expressed higher levels of procollagen type X and type II, and they concluded that PEG–GelMA–HA nano-patterned scaffolds are able to improve the chondrogenic differentiation of BMP-2-induced DPSCs efficiently. Cell-derived ECM provided a favorable microenvironment for MSC differentiation into chondrocytes in early studies [56]. Heparin is well-known for its highly sulfated glycosaminoglycan that binds to a variety of growth factors, such as vascular endothelial growth factor, transforming growth factor beta 1 (TGF-β1), and fibroblast growth factor [57]. TGF-β1 enhances chondrocyte differentiation early on by modulating proliferation, increasing alkaline phosphatase activity, and increasing proteoglycan synthesis [58]. TGF-1 can be released in a sustained manner on human fibroblast-derived ECM–hep for up to 28 days, according to Noh et al. [31] indicating that the interaction between heparin and TGF-1 is safe and effective. Some studies have found that incorporating heparin into hydrogels can improve the chondrocyte phenotype or promote the re-differentiation of dedifferentiated chondrocytes [59]. These authors proposed that heparin’s chondrogenic activity is due to the intrinsic nature of heparin, which secures endogenous growth factor (i.e., TGF-β) secretion from cells, which can enhance chondrogenesis. Guillaume et al. [34] proved for the first time that the presence of a micro scaffold has no effect on the ability of cells to form spheroids or their viability. The study also revealed that the design of the micro scaffolds was chosen on the basis of the chemical structure of fullerene, known as buckyball (BB), because it has a spherical structure and is highly porous with thin struts (i.e., 35 m), which allows a cell suspension to form a spheroid within its core. Based on these findings, these researchers demonstrated that when adipose-derived stem cell spheroids are cultured within BBs, the spheroids retain their differentiation potential (i.e., chondro-genic and osteogenic). Matrilin-3 is an adaptor protein that belongs to the non-collagenous ECM protein family. It is essential for skeletal development, including mesenchymal differentiation, chondrocyte hypertrophic differentiation, dedifferentiation, and bone mineral density maintenance [60,61]. The authors [36] used methacrylated hyaluron to encapsulate and construct scaffolds containing Ad-MSCs and matrilin-3. As a result of the 3D culture method using scaffolds, matrilin-3 was found to play a significant role in modulating the therapeutic effect of Ad-MSCs on cartilage regeneration and hypertrophy suppression.

**Figure 3:**
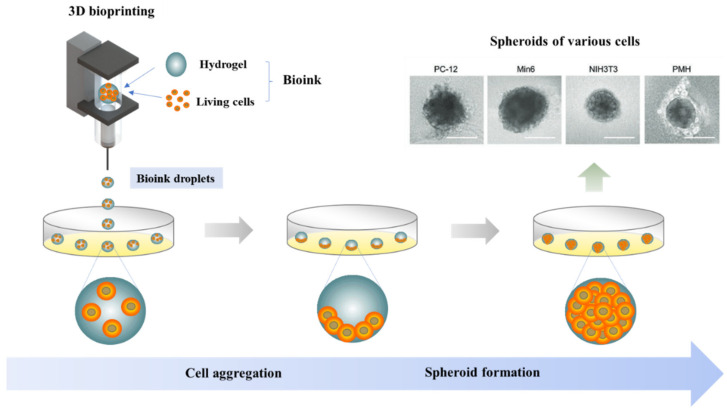
Cell-based 3D bioprinting technology using hydrogel bioinks for spheroid formation. PC-12, Neuronal cells; Min 6, pancreatic b-cells; NIH3T3, fibroblast; PMH, primary mouse hepatocytes. (Reprinted/adapted with permission from Ref. [63]. Copyright 2020, John Wiley and Sons).

Recently, 3D-bioprinting technology studies for spheroids were performed, and this technology has been used in tissue regeneration. As a field of 3D printing, 3D bioprinting technology, which integrates cells and growth factors into bio-ink and prints them in addition to structural biomaterials, is known as a new technology for soft tissue and complex tissue reconstruction [62]. Using a three-axis stage and controlling the pneumatic pressure, microextrusion-based 3D bioprinting technology has allowed for the formation and precise positioning of multiple cell spheroid types within a 3D structure (Figure 3). More significantly, it ensures that spheroid size and position are precisely controlled at the micrometer scale [63]. Yejin Park et al. demonstrated that a spheroidal bioink form was maintained immediately after printing, independent of cell type, and that using tissue-specific dECM improved cell maturation by encouraging cell-to-cell and cell-to-ECM interactions [64]. Furthermore, BugraAyan et al. indicated that a dual-layered osteochondral interface could be bioprinted using a newly developed aspiration-assisted bioprinting (AAB) technique, the first time that scaffold-free bioprinting was used in osteochondral interface engineering [65]. We suggest that the new 3D bioprinting printing technology can manufacture more precise and stable spheroids than previously possible, enhancing cartilage regeneration possibilities.

### 3.2. Effect of Exosomes on Chondrogenic Differentiation

**Figure 4:**
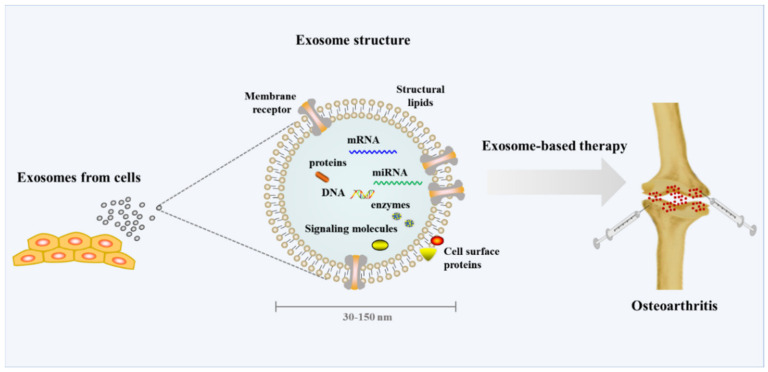
Exosome-based stem cell therapy for osteoarthritis.

Many studies have recently examined the potential use of exosomes as diagnostic markers and gene carriers for therapeutic purposes [66]. Exosomes (30–150 nm in diameter) are divided into three types: EVs’ micro-vessels/heading particles and apoptotic bodies (≥100 nm) [67]. The term “exosome” was chosen by Johnstone because “the process appeared to be akin to reverse endocytosis, with internal vesicular contents released as opposed to external molecules internalized in membrane-bound structures” [68]. Exosomes form by sprouting as intraluminal vesicles within late endosomes or multivesicular bodies’ luminal space [69]. Once multivesicular bodies are incorporated into the cellular membrane, intraluminal vesicles are secreted as exosomes. They facilitate short- to long-distance intercellular communication by transporting bioactive molecules such as DNA fragments, mRNAs, non-coding RNAs, lipids, and proteins [70]. A lot of studies have shown that MSC-derived exosomes (MSC-exos) have inherent therapeutic potential due to their intrinsic cargo. MSC-exos have demonstrated excellent efficacy in tissue repair and reconstruction in a variety of organs [71]. Exosomes have been widely used by researchers for the treatment of autoimmune and inflammatory diseases such as osteoarthritis, because they play critical roles in the modulation of inflammation and immune responses (OA) (Figure 4) [66]. However, large quantities of MSC-exos are required for preclinical and clinical research on exosome-based therapy. To achieve the desired biological outcomes, a dose of 20–200 g per mouse (109–1011 particles) is usually required. One patient required approximately 100 g/kg of exosomes at every treatment during clinical testing. Meanwhile, unlike immortal cell lines, the expansion of MSCs in culture is limited, with most studies recommending that only cells from passage six be used [71].

Other researchers used spheroid culture, scaffolds, or micro-carrier-based 3D culture to produce more exosomes than 2D cultures in recent years. This is primarily because the 3D system’s external space fills with serum-free medium, and the supernatant can be collected continuously for 3D-exosome extraction [71]. Haraszti et al. reported that, when compared to conventional 2D culture, 3D culture yielded more exosomes and highly enhanced exosome collection efficiency [72]. Moreover, Hosseinzadeh et al. [73] showed that MSCs treated with 50 and 100 g/mL MSC-EVs secreted more chondrogenic specific markers, particularly COL II, and secreted more glycosaminoglycans and proteoglycans than chondrocyte–EVs. Based on these findings, they demonstrated that EVs derived from chondrocytes and MSCs could improve MSC chondrogenesis in a cell micromass culture induced by TGF signaling. MSCs’ biological and therapeutic effects are attributed primarily to paracrine mechanisms involving the secretion of growth factors, chemokines, cytokines, and extracellular EVs. EVs may be the most valuable therapeutic agents among these paracrine molecules [74,75]. Cosenza et al. [76] showed that human umbilical cord Wharton’s jelly MSC-exos can promote the migration and proliferation of bone marrow-derived MSCs as well as chondrocyte proliferation. The authors also discovered that human umbilical cord Wharton’s jelly MSC-exos promoted macrophage polarization towards the M2 phenotype. Jin et al. [77] demonstrated that BM–MSC-exosomes could keep chondrocytes alive by increasing COLII synthesis and inhibiting IL-1-induced senescence and apoptosis. Table 2 presents the chondrogenic markers related to exosome studies.

## 4. MSC-Derived Exosome Approaches to Chondrogenic Differentiation for Osteoarthritis (OA)

One of the most general musculoskeletal diseases, osteoarthritis (OA), is distinguished by synovial inflammation, subchondral bone sclerosis, cartilage degradation, ligament calcification, and osteophyte formation [79]. Because of their inherent regenerative capacity for self-renewal and chondrogenic differentiation, MSCs have recently been used in a variety of cell therapies. However, MSC-based cellular approaches have some technical limitations, such as dedifferentiation during MSC expansion, decreased regeneration efficacy after administration, and inconsistency in large-scale cell production. To overcome these drawbacks, exosome-mediated cartilage tissue regeneration has been studied. Exosomes could be used as alternative therapeutic agents for OA treatment because they are derived from MSC transport and deliver multiple cellular components from their original MSC sources [80]. MSC-derived exosomes have been shown to protect cartilage and bone from degradation in OA by enhancing the expression of chondrocyte markers such as COLII and ACAN, decreasing catabolic markers such as MMP-13 and disintegrin and metal-loproteinase with thrombospondin motifs 5 (ADAMTS5), reducing inflammatory markers such as iNOS, protecting chondrocytes from apoptosis, and inhibiting macrophage activation [76]. OA was previously thought to be the most common aging challenge, caused by dysregulation of COLII and ACAN levels or upregulation of COLII levels [81]. COLII is the most common type of hyaline found in all cartilage tissues, and it is responsible for the stability and biological functions of healthy articular cartilage [82]. Fazaeli et al. [78] showed that while COLII expression was lower in both exosome-treated groups than in the control group, it was significantly higher in the sham and OA groups, with a higher expression in the bone-marrow-exosome group than in the adipose-tissue-exosome group. Cosenza et al. [76] found that injecting MSC-derived exosomes into the articular cavity protected mice from OA. The authors proposed that MSC exosomes could be a better therapeutic option for osteoarthritis and have potential as an alternative to cell-based approaches.

## 5. Conclusions

Many studies have indicated that the 3D culture system has enabled the efficient production of MSC-derived exosomes with enhanced therapeutic potential for diseases. Exosomes derived from MSCs have a capability in the induction of chondroprogenitor cells to proliferation and terminal differentiation into mature chondrocytes. These MSC exosomes can effectively repair cartilage by increasing chondrocyte proliferation, inhibiting apoptosis, and regulating the immune response. However, more studies are needed to determine the exact mechanisms of exosomes during cartilage repair. Previous 3D culture techniques have uneven cell densities and unstable cell cultures. 3D bioprinting technology has the advantage of enabling the in situ formation and uniform cell density of multiple cell spheroid types. Although 3D culture increased the number of exosomes, the intravehicular cargo content and function of exosomes are still unknown, and current elucidation of the role of exosome during chondrocyte differentiation by 3D culture techniques still has great limitations. Furthermore, there is a lack of understanding about the molecular signal network that mediates the improved MSC spheroid properties or cell proliferation. More molecular mechanistic studies are required to better understand and optimize MSC spheroids for clinical applications in OA.

## Figures and Tables

**Table 1:** Types of 3D cultures by cell line for chondrogenic differentiation.

Cell Lines		3D Culture Method	Finding	Ref.
Cell	Origen			
ADSCs	Human	Spinner culture	In comparison to monolayer culture, hADSCs in a spheroid culture technique showed improved in vitro chondrogenic differentiation and in vivo cartilage production.	[24]
ADSCs	Human	Magnetic levitation	MNP clearly improved GAG deposit for all cell forms, implying that MNP could be used to increase chondrogenic shift in ADSCs.	[25]
CSPCs		Hanging drop	Off-the-shelf TE cartilage with optimally sized CSPC pellets seeded within silk scaffolds demonstrated high cartilage repair capacity.	[26]
BMSCs	Rat	Hanging drop	Raw materials in the medium could be a promising route to producing cost-effective chondromimetic tissue for cartilage regeneration.	[27]
DPSCs	Mouse	Scaffold, Hydrogel	The downregulation of Nanog and EMT genes, as well as the upregulation of chondrogenic genes and the positive staining of collagen type II, indicate that nanopatterned PEG–GelMA–HA scaffolds can effectively induce DPSC chondrogenic differentiation.	[28]
MSCs		Scaffold, Hydrogel-free	The induction effect of expressed TGF-β1 results in significantly enhanced chondrogenesis of MSCs in spheroids.	[29]
MSCs	Human	Pellet culture	Spheroids derived from adipose tissue MSC had the highest concentration of ECM and glycosaminoglycans.	[30]
MSCs	Human	Scaffold, Hydrogel	TGF-β1-immobilized hFDM-hep can provide an appropriate microenvironment for hPMSC chondrogenic differentiation in 3D collagen spheroids.	[31]
MSCs	Human	Pellet culture	During spheroid culture, multiple MSC lines exhibited cell line and passage dependent aggregate morphologies that correlated highly with chondrogenic capacity.	[32]
MSCs	Human	Pellet culture	The gene ITM2A has distinct expression profiles in human primary mesenchymal stem cells derived from bone marrow and adipose tissue, and its regulation during in vitro chondrogenesis suggests that this gene may be involved in the inhibition of chondrogenesis initiation.	[33]
ASCs	Human	Scaffold, Hydrogel	When adipose-derived stem cell spheroids are cultured within BBs, the spheroids retain their differentiation potential.	[34]
MSCs	Human	Pellet culture	The SOX trio provides enough signals to induce permanent cartilage.	[35]
MSCs	Human	Scaffold, Hydrogel	Matrilin-3 plays a key role in Ad-MSC-mediated cartilage regeneration and hypertrophy suppression.	[36]

**Table 2:** Reported chondrogenic specified markers related to MSC-exosome.

Authors (Year)	Exosome Origin	Amount of Exosome	Chondrogenic Specified Markers
Hosseinzadeh et al., (2021) [73]	Rabbit bone-marrow-derived MSCs	50, 100 µg/mL	COLII, GAG, proteoglycan
Cosenza et al., (2017) [76]	Murine bone-marrow-derived MSCs	12.5, 125, 1250 ng/mL	COLII, ACAN
Jin et al., (2021) [77]	Rat bone-marrow-derived MSCs	100 µg/mL	COLII, MMP13, ADAMTS5
Fazaeli et al., (2021) [78]	Human adipose- or bone-marrow-derived MSCs	100 µg/mL	COLI, SOX9, COLII, ACAN

## Data Availability

All data used in this paper are contained within the article.
